# Supplementary material for: DENND6A links Arl8b to a Rab34/RILP/dynein complex, regulating lysosomal positioning and autophagy
Source: Nat Commun. 2024 Jan 31;15:919. doi: 10.1038/s41467-024-44957-1 (PMC10830484; doi:10.1038/s41467-024-44957-1)
Supplement: Supplementary file 1 — Supplementary Information File [file 41467_2024_44957_MOESM1_ESM.pdf]

Supplementary information

DENND6A links Arl8b to a Rab34/RILP/dynein complex, regulating lysosomal positioning and autophagy

Running title: DENND6A regulates lysosomal positioning and autophagy

Rahul Kumar*, Maleeha Khan[1], Vincent Francis[1], Adriana Aguila, Gopinath Kulasekaran, Emily Banks, and Peter S. McPherson*

Department of Neurology and Neurosurgery, Montreal Neurological Institute (the Neuro), McGill University, Montreal, Quebec, Canada.

[1]Equal contributions

*To whom correspondence should be addressed:
Email: rahul.kumar@mail.mcgill.ca; peter.mcpherson@mcgill.ca

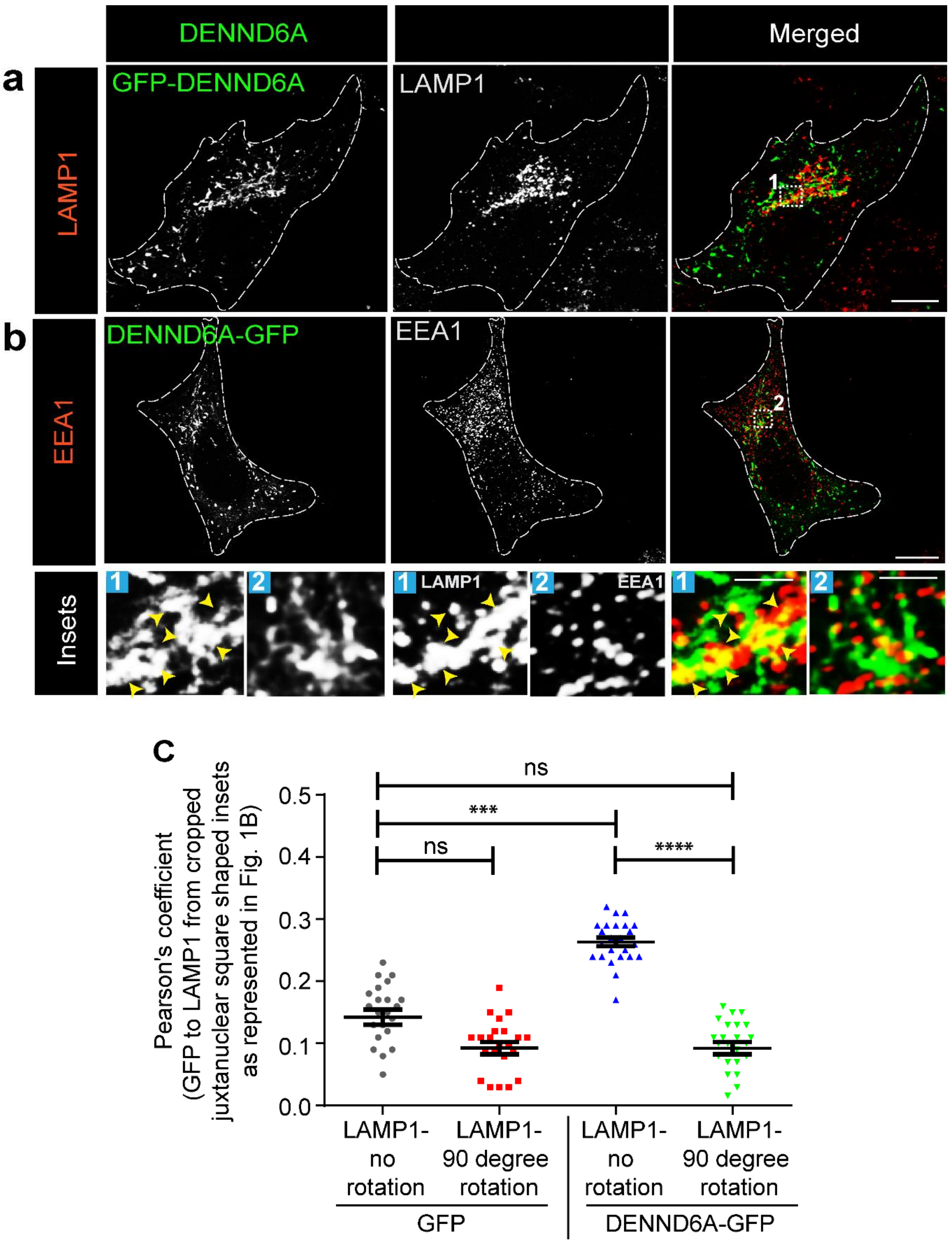

Supplementary figure 1: GFP-DENND6A localizes to lysosomes. (a) HeLa cells expressing GFP-DENND6A were fixed, stained with LAMP1 antibody and imaged using confocal microscopy (Leica SP8). The cell periphery is outlined by a white dotted line. Scale bar = 10 and 2.92 µm for low and high (inset 1) magnification. The yellow arrow indicates overlapping regions between DENND6A-GFP/LAMP1. **(b)** HeLa cells expressing DENND6A-

GFP were fixed, stained with EEA1 antibody and imaged using confocal microscopy (Leica SP8). The cell periphery is outlined by a white dotted line. Scale bar = 10 and 1.67 µm for low and high (inset 2) magnification. **(c)** Quantification of the Pearson correlation coefficient for the co-localization of GFP with LAMP from cropped juxtanuclear square shaped insets as represented in Figure 1B before and after rotating LAMP1 channel by 90 degrees; means ± SEM; Kruskal-Walis test (*** P ≤ 0.0005; **** P ≤ 0.0001; ns = not significant; n = 23 or 24 cells corresponding to GFP or DENND6A-GFP expressing cells from 3 replicates).

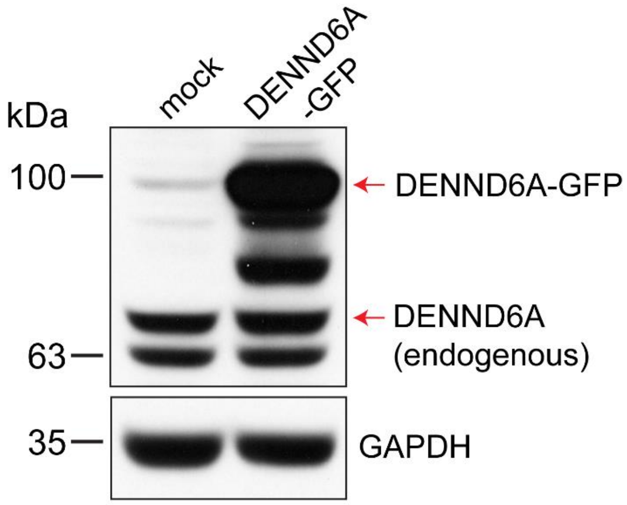

Supplementary figure 2: Levels of overexpression of DENND6A-GFP. Immunoblot showing level of overexpression of DENND6A-GFP in HeLa cells. Immunoblot probed with anti-DENND6A antibody and anti-GAPDH antibody.

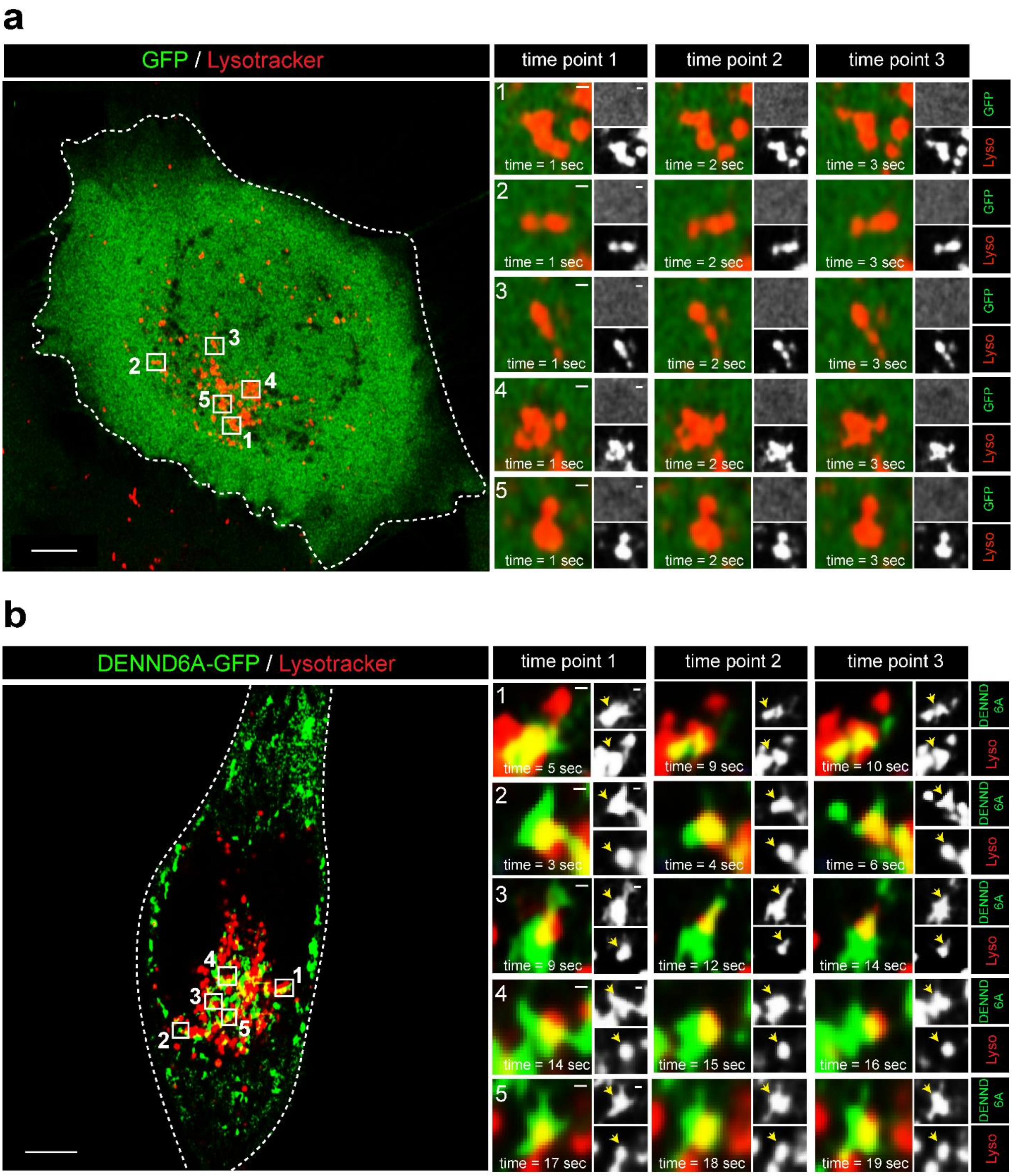

Supplementary figure 3: DENND6A-GFP localizes to lysosomes in live-cells. (a) HeLa cells transfected with GFP (green) were stained with lysotracker (red) and imaged live using LSM880 (airyscan). The cell periphery is outlined by a white dotted line. Scale bar = 5 and 0.2 µm for low and high magnification images. **(b)** HeLa cells transfected with DENND6A-GFP (green) were stained with lysotracker (red) and imaged live using LSM880 (airyscan). The cell periphery is outlined by a white dotted line. Scale bar = 5 and 0.2 µm for low and high magnification images. The yellow arrow indicates overlapping regions between DENND6A-GFP and LAMP1.

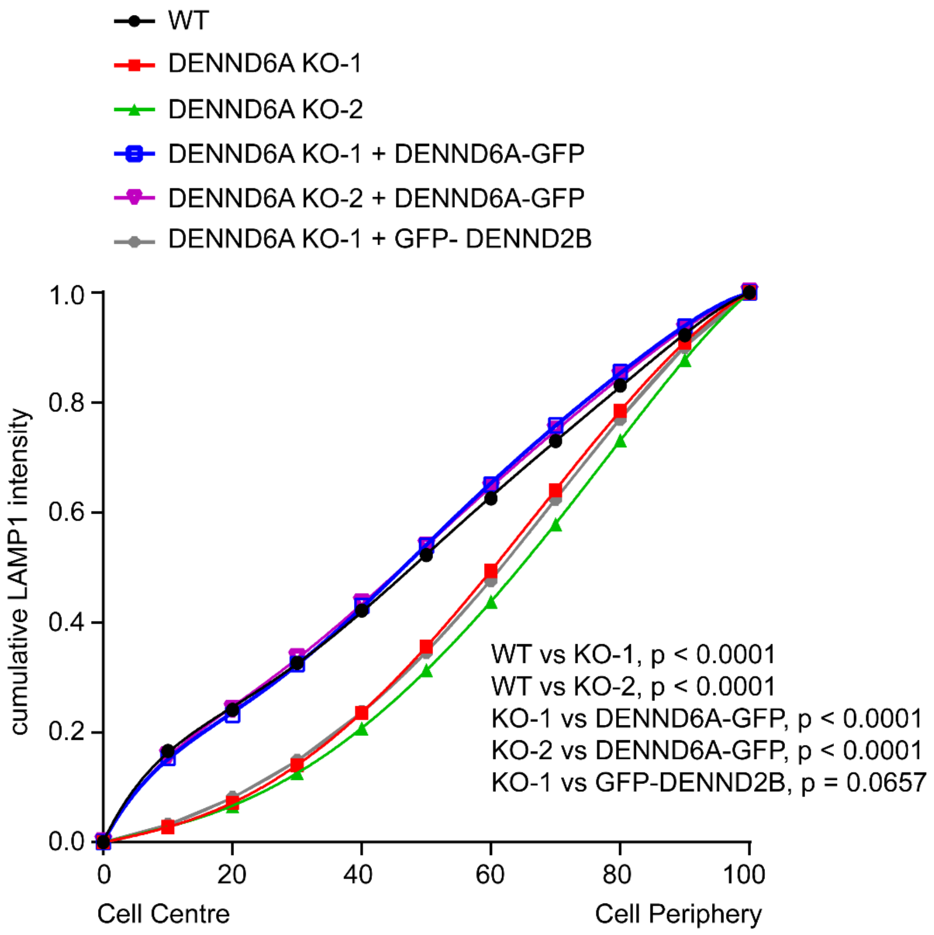

Supplementary figure 4: Loss of DENND6A causes lysosomal dispersion. Quantification of cumulative distribution of LAMP1 intensity in different indicated conditions; mean ± SEM; extra sum of F-squares test following nonlinear regression and curve fitting; n = 30, 30, 30, 27, 30, and 22 cells corresponding to WT, KO1, KO2, KO1+DENND6A-GFP, KO2+DENND6A-GFP and KO1+GFP-DENND2BD from 3 replicates.

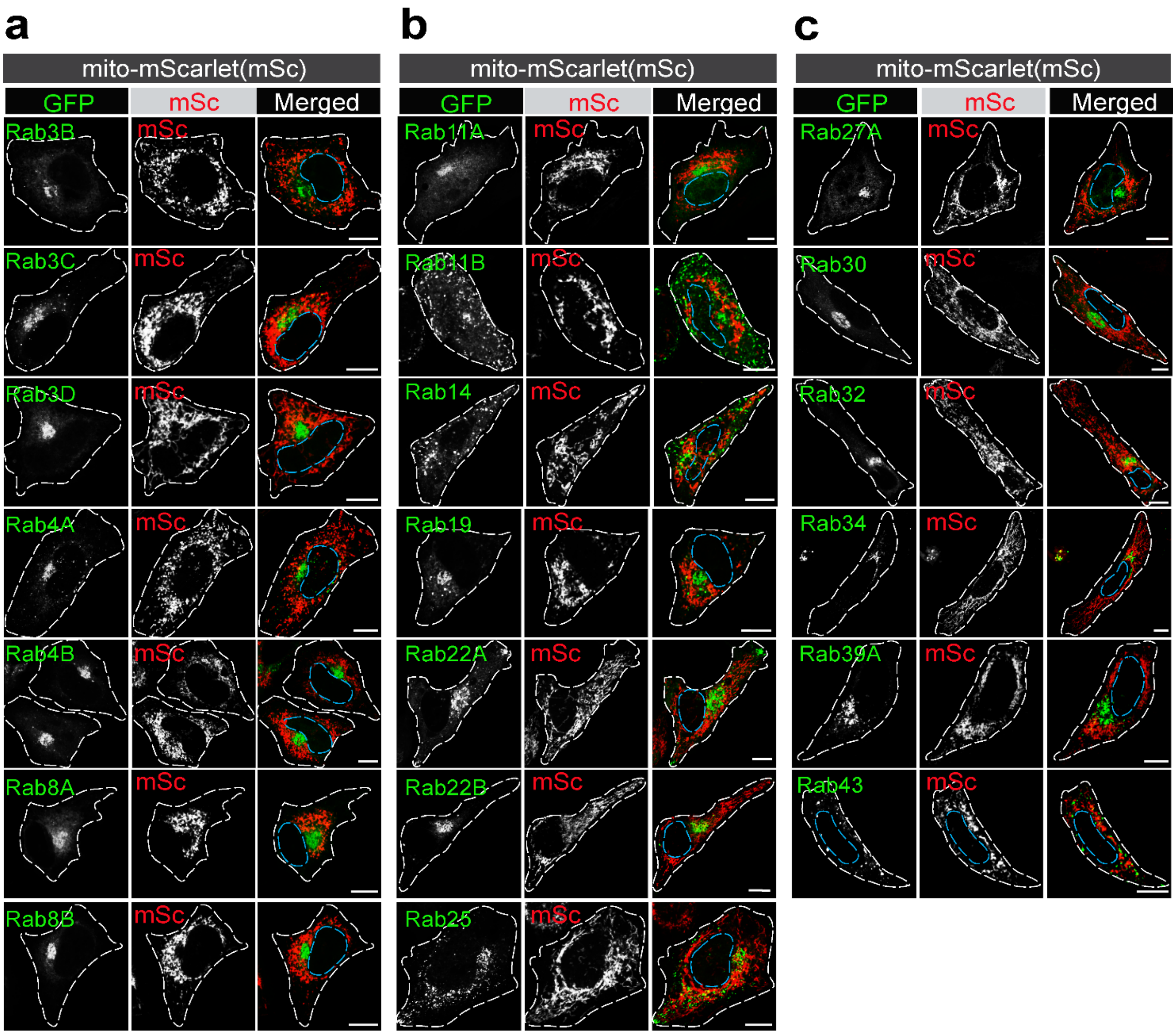

Supplementary figure 5: mScarlet(mSc) targeted to the mitochondria does not recruit Rab GTPases. (a-c) HeLa cells co-transfected with GFP-Rabs and mito-mSc were fixed and imaged. The nucleus and cell periphery are outlined by blue and white dotted line respectively. Scale bar = 10 µm.

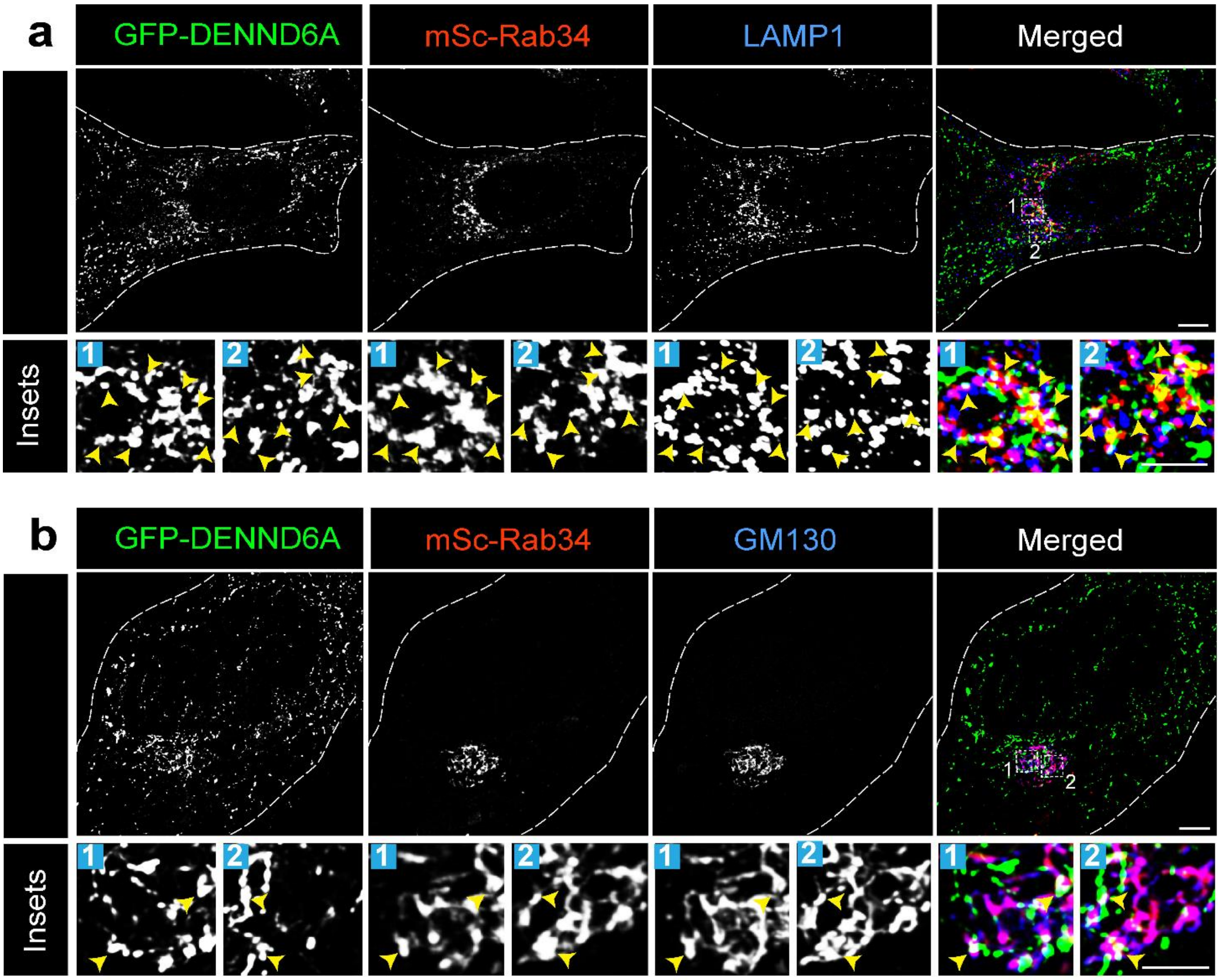

Supplementary figure 6: DENND6A/Rab34 overlaps with lysosomes. (a) HeLa cells expressing DENND6A-GFP and mScarlet (mSc)-Rab34 were fixed, stained with LAMP1 antibody. 3D-SIM images were acquired using LSM880-Elyra PS1 super-resolution microscopy. The cell periphery is outlined by a white dotted line. Scale bar = 5 and 2.25 µm for low and high magnification images. The yellow arrow indicates overlapping regions between DENND6A-GFP/Rab34/LAMP1. **(b)** HeLa cells expressing DENND6A-GFP and mScarlet (mSc)-Rab34 were fixed and stained with GM130 antibody. 3D-SIM images were acquired using LSM880-Elyra PS1 super-resolution microscopy. The cell periphery is outlined by a white dotted line. Scale bar = 5 and 2.25 µm for low and high magnification images. The yellow arrow indicates overlapping regions between DENND6A-GFP/Rab34/GM130.

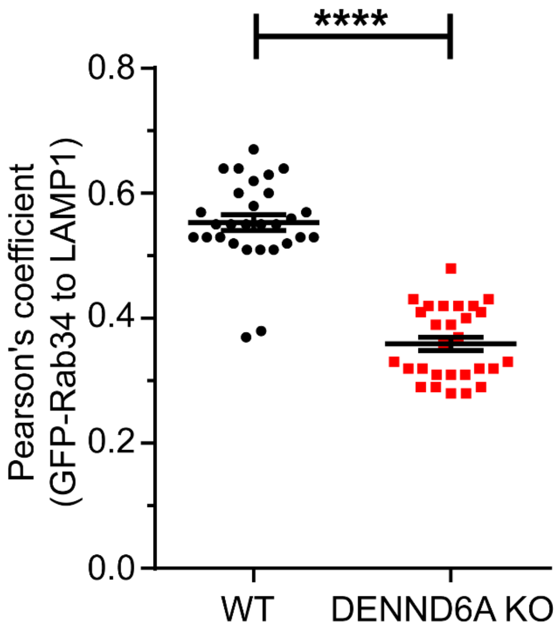

Supplementary figure 7: Loss of DENND6A reduces Rab34 localization to lysosomes. Quantification of the Pearson correlation coefficient for the co-localization of GFP-Rab34 with LAMP1 in WT versus DENND6A KO cells; means ± SEM; two-tailed Mann-Whitney U tests (**** $P \leq 0.0001$; n = 29 and 28 for WT and KO cells from 3 replicates).

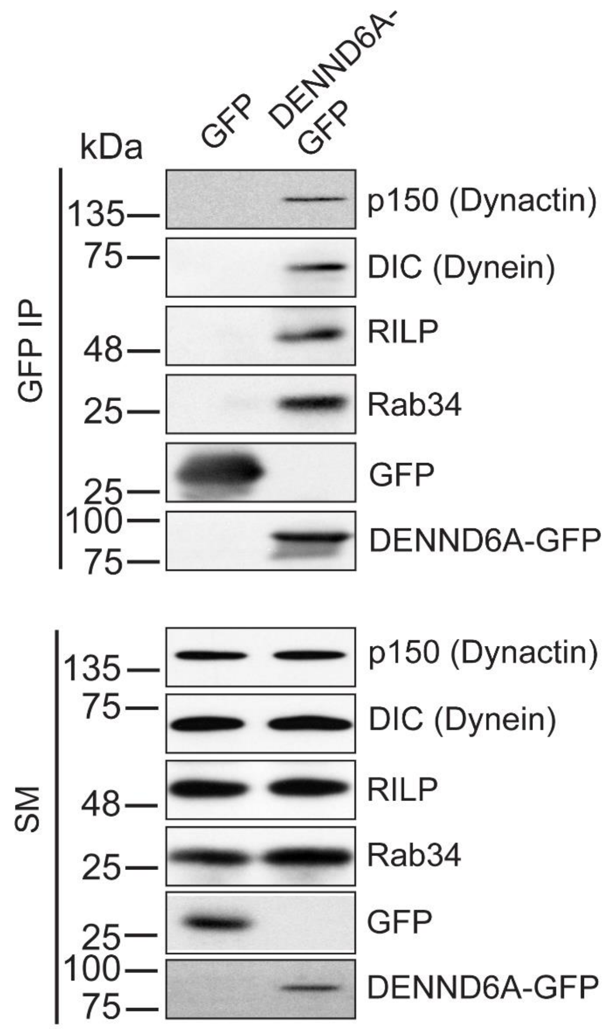

Supplementary figure 8: DENND6A coimmunoprecipitates RILP, Rab34, dynein and dynactin (p150) in a complex. HEK-293T cells were transfected with either GFP or DENND6A-GFP. At 24 hours post-transfection, cells were lysed and incubated with protein A–agarose with anti-GFP antibody (GFP IP). Specifically bound proteins were detected by immunoblot with anti-GFP antibody to detect DENND6A, anti-Rab34 antibody to detect Rab34, anti-DIC (dynein intermediate chain) antibody to detect dynein, anti-RILP antibody detecting RILP, and anti-p150 antibody to detect dynactin subunit p150 (glued).

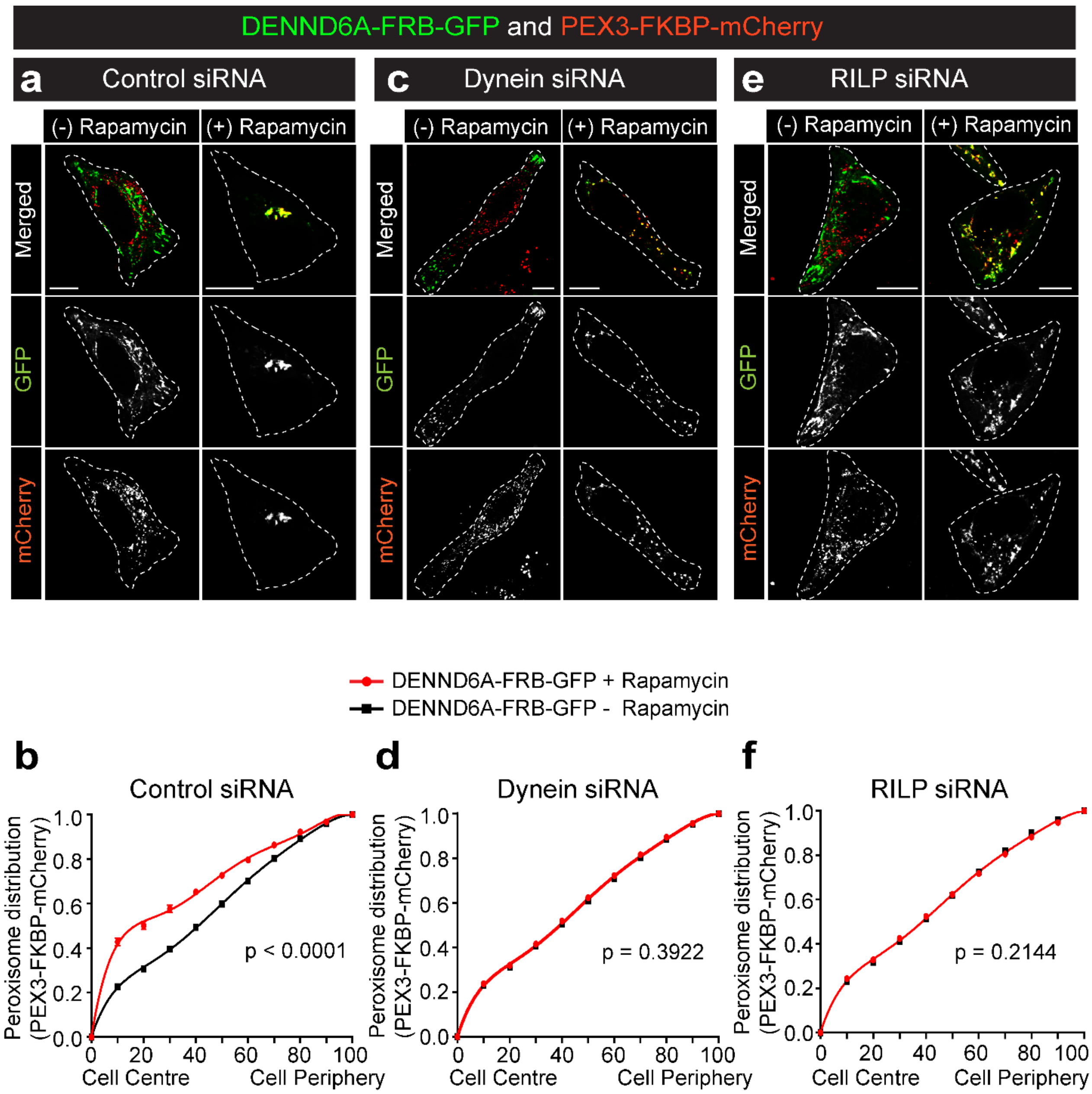

Supplementary figure 9: DENND6A mediated juxtanuclear clustering of peroxisome depends on dynein and RILP. (a, c, e) HeLa cells were co-transfected with DENND6A-FRB-GFP and PEX3-FKBP-mCherry. Subsequently, they were transfected with control, dynein, or RILP siRNA and 16 hour post-transfection treated with or without rapamycin for 1 hour. Following rapamycin treatment, cells were fixed and imaged. The cell periphery is outlined by a white dotted line. Scale bars = 10 µm. **(b, d, f)** Quantification of cumulative peroxisomal distribution (mCherry intensity) from experiments performed in **a, c** and **e**; mean ± SEM; extra sum of F-squares test following nonlinear regression and curve fitting; n [(control siRNA- rapamycin), (control siRNA+ rapamycin), (dynein siRNA- rapamycin), (dynein siRNA+ rapamycin), (RILP siRNA- rapamycin), (RILP siRNA+ rapamycin)] = (30, 30, 30, 28, 30, 30) cells from 3 replicates.

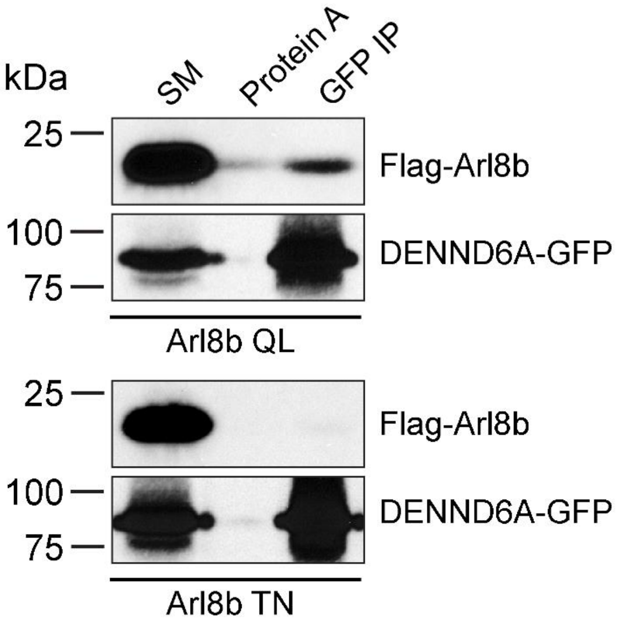

Supplementary figure 10: DENND6A binds preferentially to active Arl8b (Arl8b QL). HEK-293T cells were cotransfected with DENND6A-GFP and Arl8b QL-Flag or Arl8b TN-Flag. At 24 hours post-transfection, cells were lysed and incubated with protein A–agarose alone (mock) or protein A–agarose with anti-GFP antibody (GFP IP). Specifically bound proteins were detected by immunoblot with anti-GFP antibody to detect DENND6A or anti-Flag antibody recognizing Arl8b QL / Arl8b TN.

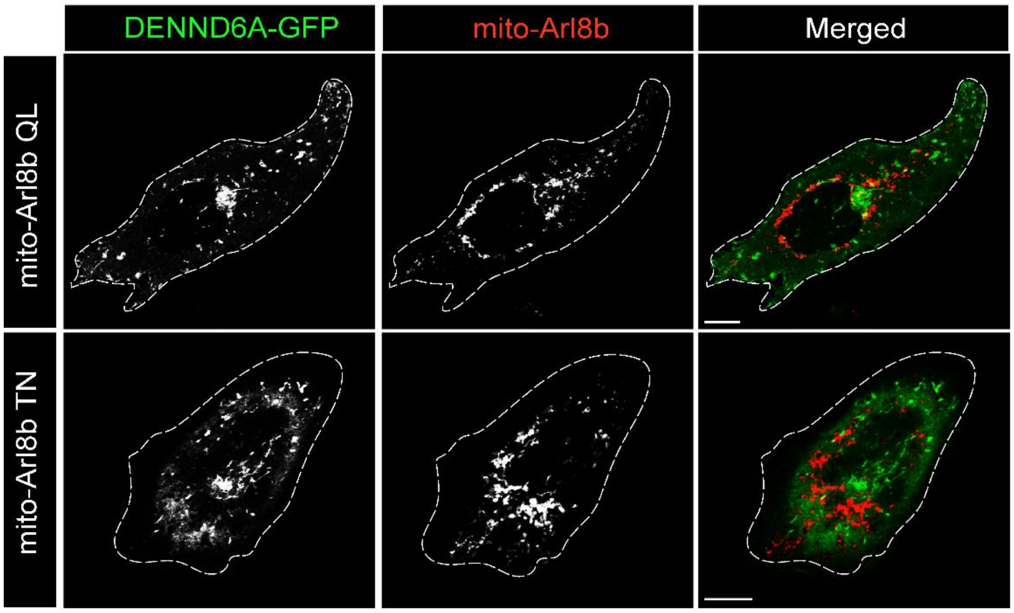

Supplementary figure 11: mito-mScarlet(mSc)-Arl8b QL targeted to the mitochondria does not recruit DENND6A. HeLa cells co-transfected with DENND6A-GFP and mito-mSc-Arl8b QL were fixed and imaged. The cell periphery is outlined by white dotted line. Scale bar = 10 µm.

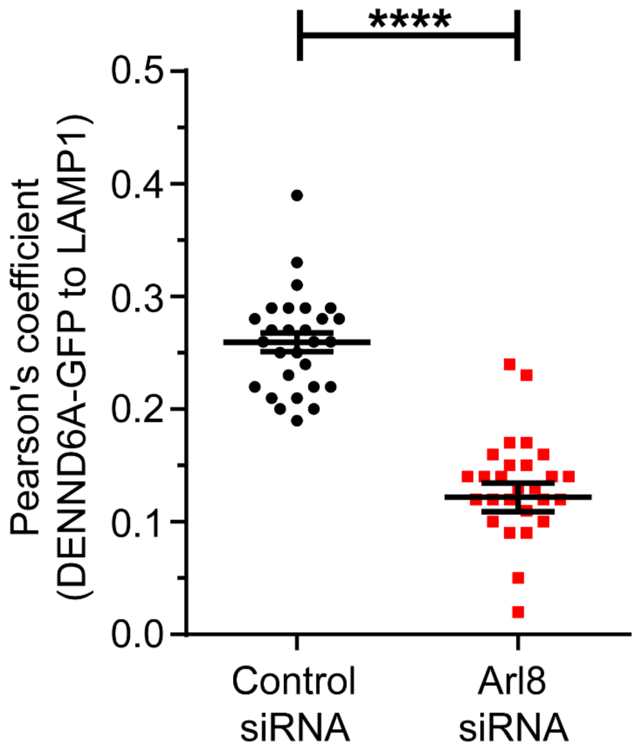

Supplementary figure 12: Loss of Arl8 reduces DENND6A localization to lysosomes. Quantification of the Pearson correlation coefficient for the co-localization of DENND6A-GFP with LAMP1 in control versus Arl8 siRNA treated cells; means ± SEM; two-tailed Mann-Whitney U tests (**** $P \leq 0.0001$; $n = 28$ for each condition, from 3 replicates).

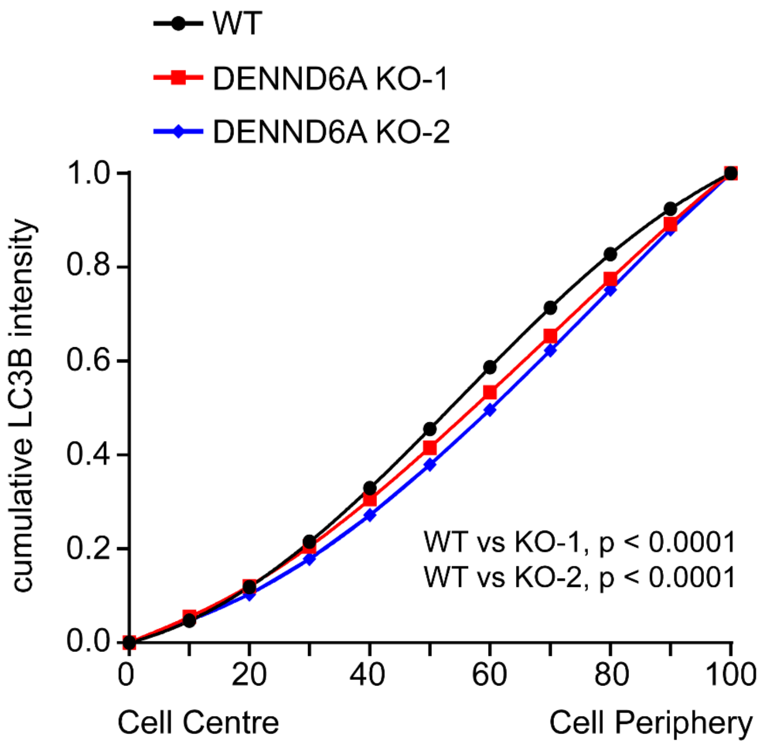

Supplementary figure 13: Loss of DENND6A impacts LC3B distribution. Graphical representation of cumulative distribution of LC3B intensity in HeLa WT, DENND6A KO-1 and DENND6A KO-2 (under unstarvaed condition); mean ± SEM; extra sum of F-squares test following nonlinear regression and curve fitting; n = 30 cells each, corresponding to WT, DENND6A KO-1 and DENND6A KO-2, from 3 replicates.

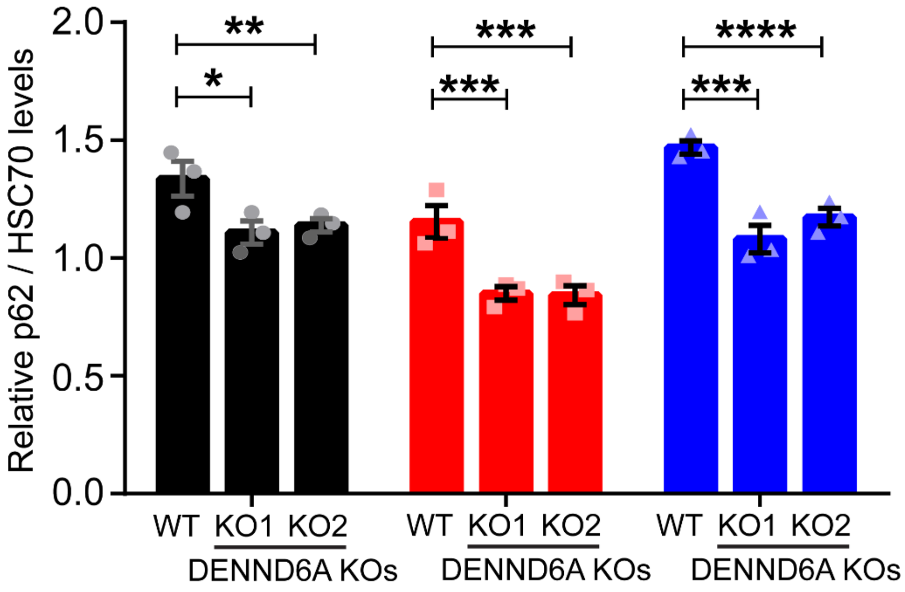

Supplementary figure 14: Absence of DENND6A reduces p62 levels. Quantification of p62 protein levels under various conditions (unstarved; EBSS; EBSS + BafA1); means ± SEM; two-way ANOVA (* P < 0.05; ** P ≤ 0.0025; *** P ≤ 0.0005; **** P ≤ 0.0001; n = 3).

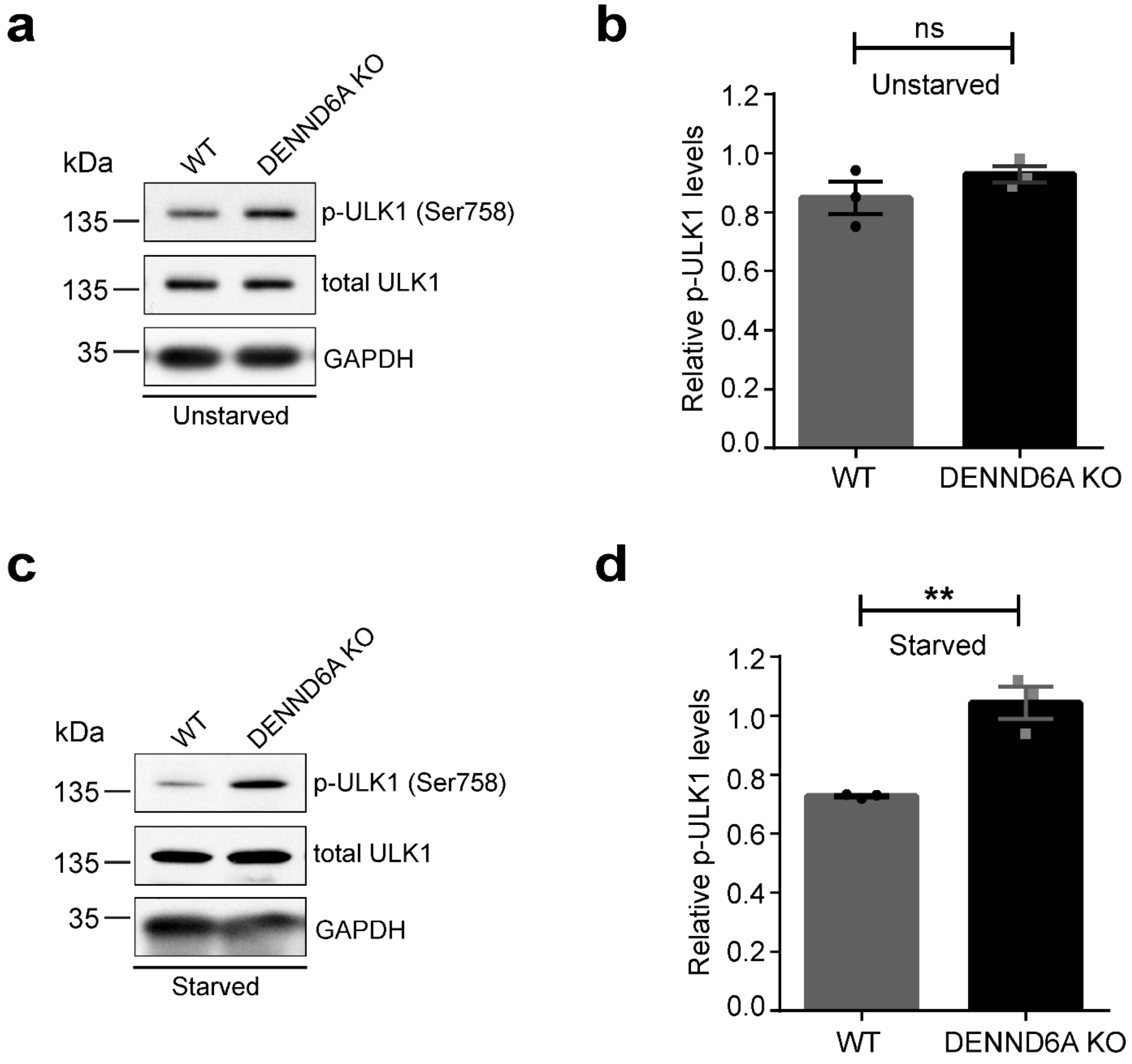

Supplementary figure 15: Loss of DENND6A inhibits starvation mediated dephosphorylation of ULK1. (a) Immunoblot showing phospho-ULK1 (p-ULK1) protein levels in HeLa WT and DENND6A KO cells under unstarved condition. Immunoblot probed with anti-p-ULK1, anti-ULK1, and anti-GAPDH antibodies. **(b)** Quantification of experiment in A; means ± SEM; two-tailed unpaired t test (ns, not significant; n = 3). **(c)** Immunoblot showing phospho-ULK1 (p-ULK1) protein levels in HeLa WT and DENND6A KO cells under starved condition. Immunoblot probed with anti-p-ULK1, anti-ULK1, and anti-GAPDH antibodies. **(d)** Quantification of experiment in C; means ± SEM; two-tailed unpaired t test (** $P < 0.0044$; n = 3).

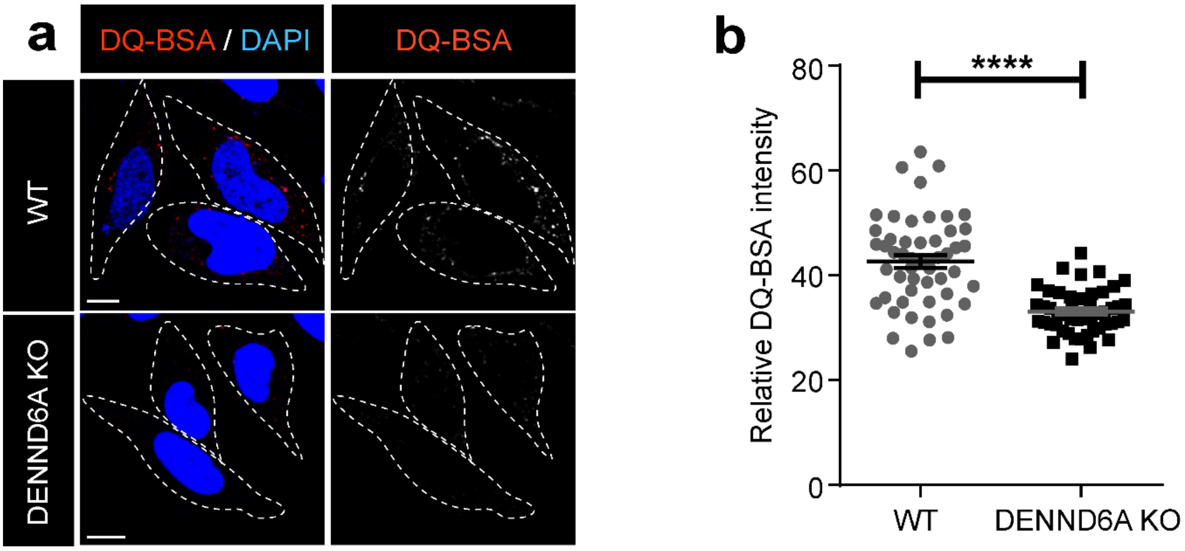

Supplementary figure 16: Loss of DENND6A impacts endocytic degradation of DQ- Red BSA. (a) HeLa WT and DENND6A KOs cells were incubated with DQ- Red BSA, washed, fixed and stained with DAPI. The cell periphery is outlined by a white dotted line. Scale bar = 10 µm. **(b)** Quantification of experiment in B; means ± SEM; two-tailed unpaired t test (**** P < 0.0001; n = 52 and 48 cells from 3 replicates, corresponding to WT and DENND6A KO).
