# Supplementary material for: DENND6A links Arl8b to a Rab34/RILP/dynein complex, regulating lysosomal positioning and autophagy
Source: Nat Commun. 2024 Jan 31;15:919. doi: 10.1038/s41467-024-44957-1 (PMC10830484; doi:10.1038/s41467-024-44957-1)
Supplement: Supplementary file 2 — Reporting Summary [file 41467_2024_44957_MOESM2_ESM.pdf]

Corresponding author(s): Peter McPherson
Last updated by author(s): Dec 18, 2023

Reporting Summary

Nature Portfolio wishes to improve the reproducibility of the work that we publish. This form provides structure for consistency and transparency in reporting. For further information on Nature Portfolio policies, see our Editorial Policies and the Editorial Policy Checklist.

Statistics

For all statistical analyses, confirm that the following items are present in the figure legend, table legend, main text, or Methods section.

n/a	Confirmed	
☐	☒	The exact sample size (*n*) for each experimental group/condition, given as a discrete number and unit of measurement
☐	☒	A statement on whether measurements were taken from distinct samples or whether the same sample was measured repeatedly
☐	☒	The statistical test(s) used AND whether they are one- or two-sided<br>*Only common tests should be described solely by name; describe more complex techniques in the Methods section.*
☒	☐	A description of all covariates tested
☐	☒	A description of any assumptions or corrections, such as tests of normality and adjustment for multiple comparisons
☐	☒	A full description of the statistical parameters including central tendency (e.g. means) or other basic estimates (e.g. regression coefficient) AND variation (e.g. standard deviation) or associated estimates of uncertainty (e.g. confidence intervals)
☐	☒	For null hypothesis testing, the test statistic (e.g. *F*, *t*, *r*) with confidence intervals, effect sizes, degrees of freedom and *P* value noted<br>*Give P values as exact values whenever suitable.*
☒	☐	For Bayesian analysis, information on the choice of priors and Markov chain Monte Carlo settings
☒	☐	For hierarchical and complex designs, identification of the appropriate level for tests and full reporting of outcomes
☐	☒	Estimates of effect sizes (e.g. Cohen's *d*, Pearson's *r*), indicating how they were calculated

Our web collection on statistics for biologists contains articles on many of the points above.

Software and code

Policy information about availability of computer code

Data collection	ZEN 2010 version 6.0.0.320 was used for LSM880-Elyra PS1 super-resolution microscopy, ZEN Black version 2.3 SP1 FP3 was used for LSM880-Airyscan microscopy, LAS X software version 3.5.5.19976 was used for Leica SP8 confocal imaging.
Data analysis	Microscopy images were analyzed using Fiji (NIH). Statistical analysis was performed using GraphPad Prism 6. Imaris version 9.7.2 was used to calculate Pearson correlation coefficient. All figures were assembled using Adobe Illustrator 2022 and Adobe Photoshop 2022.

For manuscripts utilizing custom algorithms or software that are central to the research but not yet described in published literature, software must be made available to editors and reviewers. We strongly encourage code deposition in a community repository (e.g. GitHub). See the Nature Portfolio guidelines for submitting code & software for further information.

Data

Policy information about availability of data

All manuscripts must include a data availability statement. This statement should provide the following information, where applicable:

- Accession codes, unique identifiers, or web links for publicly available datasets
- A description of any restrictions on data availability
- For clinical datasets or third party data, please ensure that the statement adheres to our policy

The manuscript and supplementary information contain all the pertinent data that underpins the findings of this study. Raw data including uncropped western blots are accessible in the accompanying Source Data file, which is included with this paper. The source data are provided with this paper.

Research involving human participants, their data, or biological material

Policy information about studies with human participants or human data. See also policy information about sex, gender (identity/presentation), and sexual orientation and race, ethnicity and racism.

Reporting on sex and gender	N/A
Reporting on race, ethnicity, or other socially relevant groupings	N/A
Population characteristics	N/A
Recruitment	N/A
Ethics oversight	N/A

Note that full information on the approval of the study protocol must also be provided in the manuscript.

Field-specific reporting

Please select the one below that is the best fit for your research. If you are not sure, read the appropriate sections before making your selection.

☒ Life sciences ☐ Behavioural & social sciences ☐ Ecological, evolutionary & environmental sciences

For a reference copy of the document with all sections, see nature.com/documents/nr-reporting-summary-flat.pdf

Life sciences study design

All studies must disclose on these points even when the disclosure is negative.

Sample size	The exact sample sizes are specified in the figure legends. Sample sizes were determined based on similar previous research (PMID: 27113757, PMID: 35314681), and three independent repeats were conducted for each experiment.
Data exclusions	We excluded any transfected cells with either very high protein expression levels and considered only those with moderate-low expression.
Replication	Experiments were replicated multiple times, as indicated in the figure legends.
Randomization	Randomization was not performed because all the cells were imaged and analyzed the same way.
Blinding	The investigator could not be blinded as they were responsible for both the preparation of the cells and analysis of the data.

Reporting for specific materials, systems and methods

We require information from authors about some types of materials, experimental systems and methods used in many studies. Here, indicate whether each material, system or method listed is relevant to your study. If you are not sure if a list item applies to your research, read the appropriate section before selecting a response.

Materials & experimental systems

n/a	Involved in the study	
☐	☒	Antibodies
☐	☒	Eukaryotic cell lines
☒	☐	Palaeontology and archaeology
☒	☐	Animals and other organisms
☒	☐	Clinical data
☒	☐	Dual use research of concern
☒	☐	Plants

Methods

n/a	Involved in the study	
☒	☐	ChIP-seq
☒	☐	Flow cytometry
☒	☐	MRI-based neuroimaging

Antibodies

Antibodies used	Mouse monoclonal Flag (M2) (F3165) antibody is obtained from Sigma-Aldrich [Western blot (WB)- 1:5000)]. Rabbit polyclonal GFP (A-6455) is obtained from Invitrogen (WB-1:5,000) , and rat monoclonal HSC70 antibody (WB-1:10,000) is obtained from Enzo (ADI-SPA-815-F). Alexa Fluor 488 (rabbit- A11088; mouse- A11001)–, Alexa Fluor 568 (rabbit- A10042; mouse- A11031)–, and Alexa Fluor

647 (rabbit- A21245; mouse- A31571)–conjugated rabbit or mouse secondary antibodies are from Invitrogen. Anti-LAMP1 mouse antibody (H4A3) is from Developmental Studies Hybridoma Bank [Immunofluorescence (IF)- 1:500, WB- 1:1000], anti-LAMP1 rabbit antibody (IF-1:200) is from Cell Signaling Technology (D2D11), anti-DENND6A antibody (PA5-66508) is from Thermo Fisher Scientific (WB-1:1000), anti-GAPDH antibody (TA802519) is from Origene (WB-1:5000), anti-HA antibody (C29F4; 3724S) is from (WB-1:1000) Cell Signaling, anti-VDAC antibody (4866S) is from Cell Signaling (WB-1:1000), anti-Catalase antibody (D4P7B; 12980S) is from (WB-1:1000) Cell Signaling , anti-Rab34 antibody (sc-376710) is from Santa Cruz Biotechnology (WB-1:1000), anti- Cytoplasmic Dynein Intermediate Chain antibody (904901) is from BioLegend (WB-1:1000), anti-Arl8a antibody (17060-1-AP) is from Proteintech (WB-1:1000), anti-Arl8b antibody (13049-1-AP) is from Proteintech (WB-1:1000), anti-LC3B antibody (3868) is from Cell Signaling (WB-1:1000; IF-1:500), anti-RILP antibody (13574-1-AP) is from Proteintech (WB-1:1000), anti-p62 antibody(MAB80281) is from R&D Systems (WB-1:1000), anti-p150 (Glued) antibody (610474) is from BD Transduction (WB-1:1000), anti-ULK1 (8054) antibody is from Cell Signaling (WB-1:1000), anti-Phospho-ULK1 (6888) antibody is from Cell Signaling (WB-1:1000), anti-GM130 antibody (610822) is from BD Transduction (IF-1:500).

Validation

Antibodies were selected in accordance with prior publications and the manufacturer's recommendations (based on their website data). All antibodies successfully detected proteins of their expected molecular weights. The specificity of the Rab34 and DENND6A antibodies used for WB was validated through knockdown and knockout experiments in this manuscript (Fig. 5i for Rab34 and Fig. 2d for DENND6A).
Flag antibody was validated by multiple publications available on the Sigma's website (such as PMID: 31417089 and PMID: 31427575).
GFP antibody was validated by multiple publications available on the invitrogen's website and has been shown to work in previous publications such as PMID: 34612142 and PMID: 36066504.
HSC70 was validated by multiple publications available on the Enzo's website and has been shown to work in previous publications such as PMID: 35196081 and PMID: 37454296.
LAMP1 antibody was validated by multiple publications available on the supplier's website (such as PMID: 35314674 and PMID: 37454104).
GAPDH antibody validated by multiple publications available on the OriGene's website (such as PMID: 37130168).
HA antibody was extensively used validated by Cell Signaling and was previously shown to work in WB such as PMID: 37803019.
RILP antibody was validated by Proteintech and also in this manuscript (FIg. 6B).
DIC antibody was validated by BioLegend and also in this manuscript (FIg. 6B).
VDAC antibody was validated by Cell Signaling and was previously shown to work in WB such as PMID: 36843552.
Catalase antibody was validated by supplier and has been shown to work in previous publications such as PMID: 35314681.
Arl8a and Arl8b antibody were validated previously (PMID: 33232665) and also in this manuscript.
LC3B antibody was validated by Cell Signaling and was previously shown to work in WB such as PMID: 25898167 and PMID: 35314681.
p150 antibody was previously shown to work in multiple publications such as PMID: 35314681 and validated by supplier.
ULK1 and Phospho-ULK1 antibody was validated by Cell Signaling and was previously shown to work in WB such as PMID: 37391422.
GM130 antibody has been validated by BD Transduction Laboratories and was previously shown to work in IF such as PMID: 36379959.
p62 antibody was validated by R&D Systems and YCharOS Inc (https://www.rndsystems.com/products/human-p62-sqstm1-antibody-2533b_mab80281).
All Alexa-Fluor secondary antibodies were validated by Invitrogen and has been shown to work in previous publications such as PMID: 35196081 and PMID: 37454296.

Eukaryotic cell lines

Policy information about cell lines and Sex and Gender in Research

Cell line source(s)	HeLa and HEK-293T cells were from ATCC (CCL-2 and CRL-1573)
Authentication	Provider's authenticated cell lines, and we also assessed cell morphology by microscopy.
Mycoplasma contamination	Cell lines were routinely screened for mycoplasma contamination using the mycoplasma detection kit (Lonza; cat# LT07-318), and all tests yielded negative results.
Commonly misidentified lines (See ICLAC register)	We did not use misidentified cell lines.
