# Supplementary material for: DENND6A links Arl8b to a Rab34/RILP/dynein complex, regulating lysosomal positioning and autophagy
Source: Nat Commun. 2024 Jan 31;15:919. doi: 10.1038/s41467-024-44957-1 (PMC10830484; doi:10.1038/s41467-024-44957-1)
Supplement: Supplementary file 4 — Description of additional supplementary files [file 41467_2024_44957_MOESM4_ESM.docx]

**DESCRIPTION OF ADDITIONAL SUPPLEMNTARY FILES DOCUMENT**

**Supplementary Movie 1**: 3D reconstruction of SIM imaging of fixed HeLa cells expressing DENND6A-GFP and stained with LAMP1 antibody. HeLa cells expressing DENND6A-GFP were fixed, stained with LAMP1 antibody. 3D-SIM images were acquired using LSM880-Elyra PS1 super-resolution microscopy. The scale bar is indicated at the bottom left corner. GFP is indicated in blue and LAMP1 is indicated in red.

**Supplementary Movie 2**: 3D reconstruction of SIM imaging of fixed HeLa cells expressing GFP and stained with LAMP1 antibody. HeLa cells expressing GFP alone fixed, stained with LAMP1 antibody. 3D-SIM images were acquired using LSM880-Elyra PS1 super-resolution microscopy. The scale bar is indicated at the bottom left corner. GFP is indicated in blue and LAMP1 is indicated in red.

**Supplementary Movie 3**: Airyscan live-cell imaging of GFP and lysotracker. HeLa cells transfected with GFP (green) were stained with lysotracker (red) and imaged live (single frame per second). The scale bar is indicated at the bottom left corner.

**Supplementary Movie 4**: Airyscan live-cell imaging of DENND6A-GFP and lysotracker. HeLa cells transfected with DENND6A-GFP (green) were stained with lysotracker (red) and imaged live (single frame per second). The scale bar is indicated at the bottom left corner.
